# Involvement of CD8^+^ T Cells in Multiple Sclerosis

**DOI:** 10.3389/fimmu.2015.00604

**Published:** 2015-11-26

**Authors:** Marion Salou, Bryan Nicol, Alexandra Garcia, David-Axel Laplaud

**Affiliations:** ^1^UMR 1064, INSERM, Nantes, France; ^2^Medicine Department, Nantes University, Nantes, France; ^3^ITUN, Nantes Hospital, Nantes, France; ^4^Department of Neurology, Nantes Hospital, Nantes, France; ^5^Centre d’Investigation Clinique, INSERM 004, Nantes, France

**Keywords:** multiple sclerosis, autoimmunity, CD8^+^ T cells

## Abstract

Multiple sclerosis (MS) is a chronic autoimmune disease of the central nervous system characterized by focal demyelination patches associated with inflammatory infiltrates containing T lymphocytes. For decades, CD4^+^ T cells have been recognized as playing a major role in the disease, especially in animal models, which has led to the development of several therapies. However, interest has recently developed in the involvement of CD8^+^ T cells in MS following the analysis of infiltrating T cells in human brain lesions. A broad range of evidence now suggests that the pathological role of this T cell subset in MS may have been underestimated. In this review, we summarize the literature implicating CD8^+^ T cells in the pathophysiology of MS. We present data from studies in the fields of genetics, anatomopathology and immunology, mainly in humans but also in animal models of MS. Altogether, this strongly suggests that CD8^+^ T cells may be major effectors in the disease process, and that the development of treatments specifically targeting this subset would be germane.

## Introduction

Multiple sclerosis (MS) is a chronic inflammatory disease of the central nervous system (CNS), resulting in disability. The clinical manifestations are very variable, and include motor, sensory, visual, and cognitive symptoms, none of them being disease specific (1). The disease is thought to be a result of both genetic and environmental factors, including infectious agents, vitamin D deficiency, obesity, and smoking (2). Neuropathological studies show disseminated patches of demyelination among the brain and the spinal cord, resulting in altered nerve conduction. Axonal loss can also be observed. Demyelinated patches are characterized by immune cell infiltration, which is absent in normal brain tissue. The infiltrate is mainly composed of macrophages, and, to a lesser extent, T and B lymphocytes (1, 3, 4).

In addition to the immune cell infiltrates, a wide range of evidence points to the pivotal role of the immune system in the development of the disease. Indeed, the genetic variants conferring a higher susceptibility to MS are associated to immune mechanisms, the animal models used for disease characterization and comprehension are autoimmune ones, and current treatments regulate or modulate the immune system (1). For decades, MS has been considered as being driven by CD4^+^ T cells, especially in animal models of the disease (1). However, treatment with monoclonal anti-CD4 antibody in phase II trials failed to reduce MS activity [measured by magnetic resonance imaging (MRI)] (5). In recent years, numerous other immune populations have been shown to be important in MS development. Among these, CD8^+^ T cells have appeared as potential major effectors within the CNS, especially in studies using human samples. In this review, we will focus on the various results that seem pivotal in understanding the involvement of CD8^+^ T cells in the pathophysiology of MS. The potential regulatory role of CD8^+^ T cells in the disease will be described elsewhere in this issue [for other reviews, see Ref. (6, 7)].

## MHC Class I Genes’ Impact on MS Risk

Numerous alleles associated with immune response have been found to be linked with increased MS risk (8–12). The strongest association, with an odds ratio of 3, was seen for human leukocyte antigen (HLA)-DRB1*15:01 in European and United States populations, which identifies CD4^+^ T cells as potent effectors in the disease (13). Major histocompatibility complex (MHC) class I alleles are also associated with MS, though to a lesser extent. HLA-A3 and B7 alleles were the first to be described as associated with a higher risk of developing the disease (14, 15). More recently, HLA A*0301 was associated with a higher risk (2×) and HLA A*0201 with a protective effect (50%) (16, 17). These associations were confirmed by Friese and colleagues in “humanized” transgenic mice for HLA*0301 and/or HLA-A*0201. These mice either developed (HLA*0301) or were protected from (HLA*0201) the disease after proteolipid protein (PLP) injection (18). In addition, these alleles are known to work in synergy with MHC class-II alleles, such as DRB1*1501, resulting in an increased risk when both are present (16). These data strongly suggest that some CD8^+^ T cells may have a beneficial or pathogenic effect, depending on the genetic background (Table 1). On top of this genetic evidence, the presence of CD8^+^ T cells in MS lesions, as well as their cytotoxic profile, evinces their involvement in the disease.

**Table 1:** **MHC-I alleles and their additive effect on MS risk with MHC-II alleles**.

MHC allele	Odds ratio
HLA-DRB1*1501	2.9–3.6
HLA-A*0201	0.52–0.7
HLA-DRB1*1501 + HLA-A*0201	1.5
HLA-A*0301	1.9–2.1
HLA-DRB1*1501 + HLA-A*0301	3.7–5.2
HLA-A*0201 + HLA-A*0301	1
HLA-B*0702	1.6–2.2
HLA-DRB1*1501 + HLA-B*0702	2.9

*MHC alleles described as implicated in MS risk are listed with their corresponding risk factor expressed by odds ratio. These data are from Fogdell-Hahn et al. and Harbo et al. (16, 17)*.

## CD8^+^ T Cells are Present and have a Pathogenic Profile in the MS CNS

### CD8^+^ T Cell Infiltration in CNS Lesions

One of the major indicators pointing toward an implication of CD8^+^ T cells in the pathophysiology of MS is the presence of these cells, in a greater number than CD4^+^ T cells, in the brain lesions of MS patients. The fact that CD8^+^ T cells outnumber CD4^+^ T cells in MS lesions was first observed in the 80s, in particular in the parenchyma, and was regardless of differing clinical parameters, such as disease duration, disease evolution, and therapy (19). In 1986, Hauser et al. studied 16 cases of progressive MS, and observed up to 50 times more CD8^+^ T cells in both the parenchyma and in perivascular cuffs of active lesions, with no case of more CD4^+^ than CD8^+^ T cells. CD8^+^ T cells also predominated in normal-appearing white matter (NAWM) (20). Recently, our study of 22 lesions in three MS patients found the same, CD8^+^ T cells being predominant regardless of the lesion type studied (21). Other studies have reached the same conclusion by single cell analysis in different MS patients (22–24). Although not all the studies concur with the above (25), others put forward the hypothesis that CD8^+^ T cells might be more prevalent in the parenchyma while CD4^+^ T cells would stay in the perivascular areas (26). Recently, in tissue block section of MS patients, CD8^+^ T cells were described as being often present in cortical plaques (54 of 70 cortical plaques analyzed). This type of plaque has been found to be associated with disease progression and cognitive impairment in the early stages of MS (27).

### MHC-I Expression and CNS Damage

**Figure 1:**
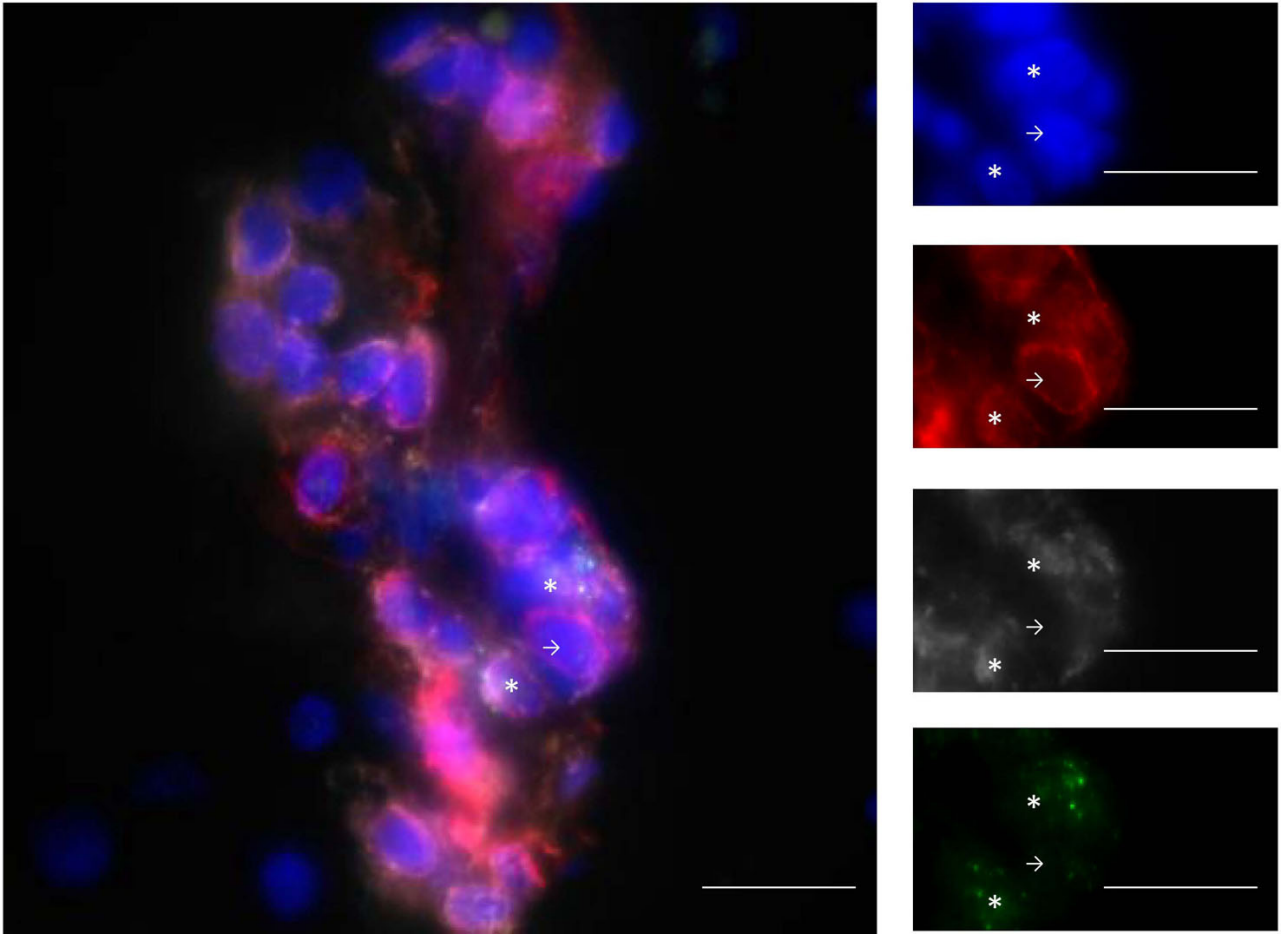
**Infiltrating T cells are mainly CD8^+^ T cells and express GZM-B**. Example of staining with DAPI (blue), CD3 (red), CD8 (gray), and GZM-B (green). The line in the pictures indicates 20 μm. Stars show CD3^+^CD8^+^GZM-B^+^ and arrows show CD3^+^CD8^−^GZM-B^−^ cells. GZM-B: granzyme-B. From personal data.

MHC-I expression and presentation is necessary for CD8^+^ T cells to carry out their cytotoxic functions. In 2004, a study including 30 MS patients and 21 controls quantified the expression of MHC-I on various cell subtypes within the CNS by immunohistochemistry and fluorescence methods (28). While constitutive expression of MHC-I on macrophages/microglia and endothelial cells was observed, MHC-I expression was gradually upregulated on astrocytes, oligodendrocytes, neurons, and axons depending on the disease type (inactive, chronic active, and active MS) and lesion activity (inactive, periplaque white matter, and active), making these cells potential targets for CD8^+^ T cells in the context of the disease. Consistent with this, CD8^+^ T cells have been shown to be able to mediate axonal transection *in vitro* (29). In this study, murine neurons induced to express MHC-I were pulsed with a dominant peptide of the lymphochoriomeningitis virus envelope glycoprotein (GP33). Five to 30 min after culture with antigen-specific cytotoxic CD8^+^ T cells, neurite breakage appeared in contact zones between CD8^+^ T cells and neurites. Confocal live imaging gave a clear image of this process. Axonal transection has also been suggested in MS (30). Indeed, axonal injury, in 88 brain biopsy samples from 42 patients, correlated with the number of CD8^+^ T cells, but not CD3^+^ T cells, found in the lesions (31). Variable proportions of lesion-infiltrating CD8^+^ T cells express granzyme B [Figure 1, personal results from Ref. (21)] and interferon γ (IFNγ), further evincing the ability of these cells to damage the CNS (21, 25, 32). In conclusion, CD8^+^ T cells seem more likely than CD4^+^ T cells to mediate CNS damage, in particular through their cytotoxic and proinflammatory properties.

### Pathogenic CD8^+^ T Cells in the CSF

Deciphering the mechanisms involved in MS development is made difficult by the limited access to the CNS compartment. As such, a lot of studies focus on the cerebrospinal fluid (CSF) as a surrogate compartment for understanding the T cell processes occurring *in situ*. In 2007, an enrichment of effector memory CD8^+^ T cells in the CSF of 52 relapsing–remitting MS patients was observed at the beginning of the disease (33). This, together with an enrichment of granzyme B-expressing CD8^+^ T cells, has been confirmed in 17 other relapsing-remitting patients (25). Interestingly, the effector memory CD8^+^ T cells had increased *in vitro* migration through a model of the blood–brain barrier, especially those producing granzyme B, perforin, IFNγ, and interleukin 17 (IL-17). This was further confirmed in a mouse experimental autoimmune encephalomyelitis (EAE) model (25). Another study found that granzyme A and B levels were higher in the CSF of patients in flare up, compared to those in clinical remission and control patients (34). Altogether, these results suggest a specific enrichment of effector memory CD8^+^ T cells in the CNS compartment in MS and place them as disease effectors.

### CD8^+^ T Cell Migration into the Brain

Studying the mechanisms leading to CD8^+^ T cell transmigration into the CNS further highlights their involvement in the disease process. Blockade of α4 integrin in EAE mice immunized with myelin oligodendrocyte glycoprotein (MOG)_35–55_ yields a decreased number of infiltrating CD8^+^ T cells, together with a reduced EAE score. However, a similar effect has been described for CD4^+^ T cells (25). More recently, melanoma cell adhesion molecule (MCAM), expressed by a subset of human effector CD8^+^ T cells, was reported to be upregulated during MS relapse compared to controls (35). Interestingly, MCAM blockade prevents the transmigration of human CD8^+^ T cells across a blood–brain barrier (BBB) model and decreases the EAE score in active, transfer and spontaneous models (36–38). As MCAM binds itself and laminin 411 (37), which are both expressed by endothelial cells, the mode of action of MCAM blockade is not yet known (35). P-glycoprotein (also known as multidrug resistance protein 1), a transporter involved in drug efflux (39) and in cytokine/chemokine secretion (40), has also been shown to be important for the trafficking of CD8^+^ T cells into the brain during the disease. Indeed, Mdr1a/b KO mice show significantly reduced EAE (40). In another study, P-glycoprotein silencing led to decreased CD8 infiltration into the brain, with no effect on CD4^+^ T cells (41). P-glycoprotein control of endothelial C–C chemokine ligand 2 (CCL2) secretion was responsible for this result. Indeed, EAE mice lacking this protein or CCL2 show significantly reduced CD8^+^ migration into the brain. More significantly, CCL2 transcript has also been found to be elevated in six MS lesions compared to six controls (41).

In conclusion, various studies on brain, spinal cord, and CSF, as well as on the mechanisms allowing T cell entry into the brain highlight CD8^+^ T cells’ potential role in the development of MS.

## CD8^+^ T Cell Repertoire and Reactivity in MS

A number of studies have focused on the T cell pools that arise with MS in order to understand their role in its pathophysiology. Different CNS and non-CNS antigens have been used to search for autoreactive T cells (3, 42), but to date the triggering antigen(s) is unknown. The search for a specific antigen is made more difficult because of the mechanisms of molecular mimicry, epitope spreading, bystander activation, and/or dual T cell receptor (TCR) (43–48). Numerous studies have analyzed the T cell repertoire, allowing the identification of reactive T cells without the need to know the recognized antigen. Indeed, after antigen recognition, T cells undergo clonal proliferation, and this expanded T cell population can be identified within the total T cell pool.

### CD8^+^ T Cell Repertoire in Lesions and in CSF

Two different groups, including our own, have studied the TCR repertoire of lesion-infiltrating T cells, either by single cell analysis (22, 23) or by whole TCR analysis using high-throughput sequencing, in comparison with sorted populations from the blood (21). These studies show that infiltrating CD8^+^ T cells are oligoclonal, i.e., only clones bearing particular TCRs specific to each patient are represented within the lesion, which is less the case for CD4^+^ T cells. These oligoclonal CD8^+^ T cells are also found in different locations in the brains of the same patients. In addition, some of these clones harbored silent mutations (23). These data strongly suggest antigen-driven selection or activation processes, and identify these infiltrating CD8^+^ T cells as disease effectors.

### CD8^+^ T Cell Repertoire Alteration in CSF and Blood

Different studies have observed that the blood of MS patients exhibits more clonal expansions than that of controls (49–53). In 35 patients, our group showed that the repertoire was more skewed in the CD8 than in the CD4 compartment, further suggesting the involvement of CD8^+^ T cells (50). The repertoire was even more skewed in the CSF than the blood of MS patients, especially in CD8^+^ T cells which interestingly expressed memory T cell markers (54–56). Of note, clonal expansions were correlated to the clinical activity of the disease. We observed more blood expansions in MS patients with an active form of the disease, assessed by MRI (57). Muraro et al. published a case study that extensively studied the TCR Vβ repertoire in the CSF of one MS patient, and described more expansions in that compartment during relapse than during remission (58). Altogether, these data strongly suggest an implication of oligoclonal T cells, especially CD8^+^ T cells, in the pathophysiology of MS.

However, whether expanded T cells in blood and CSF correlates with T cell expansion in the CNS, which is likely to participate in disease development, was unknown until recently. Indeed, only one study had suggested that some expanded CNS CD8^+^ T cell clones could persist in the blood and in the CSF for several years, seen in two MS patients (24). Very recently, we confirmed and extended this result by comparing the TCR repertoires in the blood, CSF, and CNS (22 lesions with various locations and activities) of three MS patients (21). Using CDR3 spectratyping and high-throughput sequencing, we showed for the first time that the CSF repertoire mirrors that of the CNS, which is, to a lesser extent, also similar to that of blood CD8^+^ T cells. This further evinces the involvement of CSF and blood expanded CD8^+^ T cells in MS and further identifies CSF expanded T cells as good surrogates for infiltrating T cells.

### CD8^+^ T Cell Autoreactivity

A number of studies have been looking at autoreactive T cells in MS, using different methods and antigens, but the results are hardly comparable between the studies and no consensus has been found as to the presence of CNS-specific CD4^+^ or CD8^+^ T cells in MS patients [reviewed in Ref. (42)]. In 2004, Crawford et al. adapted a flow cytometry approach to analyze antigen-specific CD4^+^ and CD8^+^ T cell proliferative response in MS patients. They showed that relapsing–remitting MS patients have a higher proportion of CD8^+^ T cell responses against CNS peptides compared to healthy volunteers (HV) or primary progressive MS patients and that this is not the case for CD4^+^ T cells (59). In another study, CD8^+^ T cells specific to apoptotic epitopes have been shown to be overrepresented in MS patients (in a cohort of 26 compared to 27 HV), and to be able to produce IFNγ and/or IL-17 when stimulated with apoptotic epitopes (60). Interestingly, these cells have been found also in the CSF and correlate with the level of disease disability, which strongly suggests their involvement in the immunopathology (60). Finally, Zang et al. tested the proliferation of CD8^+^ T cells in contact with irradiated PBMC incubated with degenerated myelin basic proteins (MBP), showing that autoreactive CD8^+^ T cells recognizing MBP proteins were increased in the blood (15 MS patients compared to 15 HV) (61). Recently, high-throughput sequencing on paired blood and CSF samples of MS patients and control gave further evidence of the implication of a particular subset of CD8^+^ T cells in MS (62), a specific enrichment of Epstein–Barr virus (EBV)-specific CD8^+^ T cells being observed in the CSF of MS patients.

These results evince the existence of a pool of CD8^+^ T cells able to damage the CNS, however the triggering events as well as the antigens recognized remain unclear, necessitating the development of other methods in order to isolate and study the relevant T cells.

## Animal Models Identify CD8^+^ T Cells as Potent Effectors in the Disease

Prior to 2001, most models focused on the involvement of CD4^+^ T cells in the development of EAE. Two groups, developed, in parallel, EAE models based on specific CD8^+^ T cell adoptive transfer. First, in mice C57BL/6, Sun et al. observed that the adoptive transfer of pMOG_35–55_ CD8^+^ T cells led to the development of an EAE that was both longer and more severe than the disease actively induced by the injection of the MOG_35–55_ peptide (63). EAE did not develop in mice lacking β2 microglobulin, further supporting the involvement of CD8^+^ T cells in the development of the disease (63). These data were confirmed in 2005, and the authors further identified the pMOG_37–46_ peptide as the minimal peptide recognized by the pMOG_35–55_-specific CD8^+^ T cells that both led to IFNγ production by these cells and to the development of severe EAE (64). In another 2001 study, Huseby et al. showed that a rapid and severe CNS-autoimmune disease could be induced in C3H mice after adoptive transfer of CD8^+^ T cells lines reacting against the MBP_79–87_ peptide (65). The severity of the disease was reduced when coinjecting anti-IFNγ with the CD8 MBP-specific cell lines, supporting a pivotal role for this cytokine in the development of the disease. Interestingly, the clinical symptoms, as well as the CNS lesion types and distributions observed, were different from those induced after CD4^+^ T cell transfer, better reflecting the clinical features of the human disease (65). The authors then proposed that the different MS clinical manifestations could be linked to the activation of either CD4^+^ or CD8^+^ T cells. Since then, CD8^+^ specific models have mainly been developed by transfer of T cells bearing TCR recognizing transgenic epitopes expressed on oligodendrocytes (66). In 2008, Saxena et al. developed a double knock-in transgenic mouse in which the influenza hemagglutinin (HA) expression was restricted to oligodendrocytes (67). The injection of *in vitro* activated CD8^+^ T cells bearing a TCR specific for HA led to CNS inflammation and demyelination. Of note, the activated CD8^+^ T cells produced granzyme B and IFNγ and exhibited cytotoxicity against cells loaded with HA *in vitro*. The authors were able to track these cells in the lesions, in close proximity to oligodendrocytes and in association with microglia activation. In another study, Na et al. showed that double transgenic mice with ovalbumin (OVA) expression in oligodendrocytes and OVA-specific TCR CD8^+^ T cells (OT-I) – but not OVA-specific TCR CD4^+^ T cells (OT-II) – develop a spontaneous EAE with demylinated and infiltrated lesions (68). The disease developed during the first 10–12 days of life, when CD8^+^ T cells still have access to the CNS, and was amplified by IFNγ. Of note, blocking the recognition of the OT-I CD8^+^ T cells with an antibody specific for the OVA-peptide/MHC-I complex prevents disease development (69). Interestingly, most of these models develop either clinical manifestations and/or infiltration characteristics that have a greater resemblance to MS symptoms and infiltrates than CD4-mediated EAE models (65, 67, 68).

Another model has been used to study the mechanisms by which CD8^+^ T cell infiltrate and damage the CNS. In Theiler’s murine encephalomyelitis virus (TMEV)-infected mice, the viral model of MS, CD8^+^ T cells secreting perforin were shown to be involved in BBB disruption and astrocyte activation (70). CD8^+^ T cells have also been shown to be able to enter the CNS in a naïve CL4 mice model without peripheral activation (CD8^+^ T cells specific for HA). Even if they remained inactivated *in situ*, they were able to proliferate when HA was injected intracerebrally. Interestingly, blocking MHC-I led to the reduction by 76% of the trafficking (71). Recently, Sobottka et al. further described mechanisms of presentation into the CNS. Using living brain slices preincubated with IFNγ (to mimic CNS inflammation) and OVA, they showed that OT-I CD8^+^ T cells (OVA-specific) were able to mediate axonal damage. Moreover, they obtained the same results using transgenic oligodendrocytes-expressing OVA in their living brain slices, making them potential target cells for CD8^+^ T cell pathogenicity (72).

Animal models depending on CD8^+^ T cells are, thus, relatively recent, and their study may shed new light on the mechanisms involving these cells in disease development (Table 2).

**Table 2:** **Summary of EAE models used to study CD8^+^ T cells**.

Model	Mode	Background	Results	Publication
WT	MOG_35–55_ CD8^+^ injection	WT (C57BL/6)	Severe and permanent EAE	(63)
	MBP_79–87_ CD8^+^ injection	WT (C3H)	Severe EAE with similarities with MS features	(65)
	MOG_35–55_ CD8^+^ injection	WT (C57BL/6)	Severe and long-term EAE	(64)
Transgenic	HA CD8^+^ injection	Oligodendrocytes Tg for HA expression (CL4)	CNS inflammation and demyelination	(67)
	OVA CD8^+^ injection	Oligodendrocytes Tg for OVA expression (C57BL/6)	Severe/lethal EAE	(68)
“Humanized” transgenic	MOG_35–55_ + MOG_181_ injection	HLA-A*0201 Tg (C57BL/6)	More severe EAE than MOG_35–55_ alone	(73)
	PLP_45–53_ injection	HLA-A*0201-2D1 TCR (specific for PLP_45–53_) double Tg (CBA/c × C57BL/6)	MS-like disease (relapsing–remitting)	(18)

*HA, hemagglutinin; OVA, ovalbumin; Tg, transgenic; MOG, myelin oligodendrocyte glycoprotein; MBP, myelin basic protein; PLP, proteolipid protein; EAE, experimental autoimmune encephalomyelitis; WT, wild type; CNS, central nervous system; MS, multiple sclerosis; TCR, T cell receptor*.

## IL-17-Producing CD8^+^ T Cells as Potent Effectors in MS

Similarly to CD4^+^ T cells, the implication of IL-17-producing CD8^+^ T cells in MS has been recently suggested. The first study suggesting this was performed on 18 frozen CNS samples from 14 MS patients (26). Seventy to 80% of the infiltrating T cells, both CD4^+^ and CD8^+^ T cells, expressed IL-17 in active and chronic active lesions, shown through double immunofluorescent staining. This percentage was dramatically lower, at 20%, in chronic inactive lesions and NAWM, suggesting significant involvement of these IL-17-producing CD4 and CD8^+^ T cells in MS pathogenesis. After *in vitro* stimulation of blood samples, another study observed more IL-17 producing CD8^+^ T cells in the 20 MS patients than in the 16 controls (74). CD8^+^ T cells secreting IL-17 after *in vitro* stimulation were present in greater frequency in the CSF than in the blood of 17 MS patients in the early stages of the disease (75). Finally, in EAE, IL-17-producing CD8^+^ T cells were found to be necessary for IL-17-producing CD4^+^ T cell accumulation in the CNS and for disease development (75).

In human, the *in vitro* production of IL-17 is restricted to CD161-expressing cells (76). These cells have been shown to be present in CNS lesions of MS patients and the majority of them produce IFNγ (IL-17 staining was not performed on these samples) (32). In addition, an enrichment of CD8^+^CD161^hi^ in the blood of MS patients has also been evidenced, suggesting a specific involvement in the disease (32).

Recently, it has been shown that more than 80% of these cells are mucosal-associated invariant T (MAIT) cells. MAIT cells are a subset of innate effector memory T cells bearing a semi-invariant TCR (Vα7.2-Jα33/12/20) in humans (77–82). They are restricted to the MHC class-I related protein I (MR1) and have antimicrobial properties both *in vitro* and *in vivo* (83–86). Although they have been correlated with various autoimmune diseases (87–90), their implication, especially in MS (32, 91, 92), remain elusive. MAIT cells are present in the CNS of MS patients, but at very low frequencies compared to in the blood (92–94). This argues against a particular implication of this IL-17-producing CD8^+^ T cell subset in the pathophysiology of MS (92, 95), similar to what has been described for psoriasis, where conventional IL-17-producing CD8^+^ T cells might be more pathogenic than MAIT cells (87).

Other IL-17-producing CD8^+^ T cell subsets have been described (96), with different markers, such as MCAM (97), but further research is necessary to decipher their involvement in MS pathogenesis.

## Multiple Sclerosis: A Close Collaboration Between CD4^+^ and CD8^+^ T Cells

Even though we are convinced that CD8^+^ T cells play a pivotal role in MS pathophysiology, it seems obvious that they interact with other subsets – especially CD4^+^ T cells – in mediating MS development. Indeed, CD8^+^ T cells may migrate first into the CNS, further allowing the infiltration of CD4^+^ T cells. This proposition is supported by the fact that a small infiltrate of CD4^+^ T cells was found in the CNS of a CD8-specific mouse model of EAE (67). Moreover, that fact that both CD8^+^ and CD4^+^ T cells produce IL-17 at the same frequency in brain lesions gives evidence for the involvement of both cell types. However, whether the damage caused by these cells is direct or bystander is still unclear. Indeed, the initiating event, which is still unknown, leads to an *in situ* inflammatory context. Amplifying inflammatory loops are likely to develop, leading to BBB disruption and to the infiltration of other immune cell subsets. The inflammatory climate and the subsequent destruction of CNS cell types result in the release of self-antigens. These antigens, usually sequestered in a compartment that is poorly accessible for immune cells, are then available for recognition and further activation of the immune compartment *in situ*. This phenomenon is well described in the EAE model. Ji et al. showed that in their EAE model, induced by the transfer of MOG-specific CD4^+^ T cells, particular dentritic cells (DC) derived from inflammatory monocytes (Tip-DC) lead to epitope spreading to MBP-specific CD8^+^ T cells *in situ* (98).

**Figure 2:**
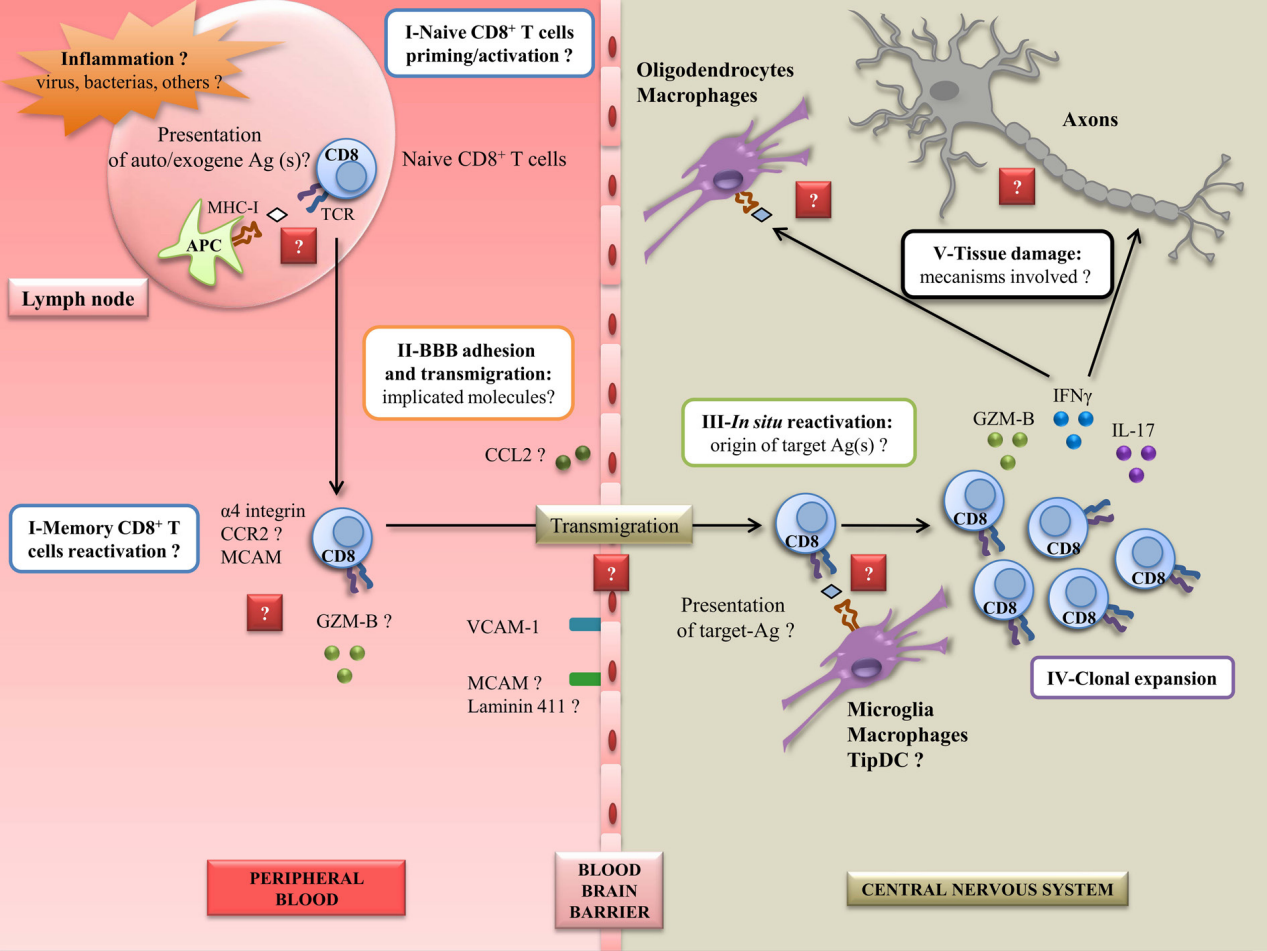
**Steps to elucidate to better understand CD8^+^ autoreactivity mechanisms in MS disease**. A peripheral inflammation induced by pathogens (such as EBV) could occur in case of uncontrolled infection. This can lead to the activation or reactivation of CD8^+^ T cells, and the expression of several molecules implicated in adhesion, migration, and cytotoxicity, currently not well characterized. In this inflamed state, the BBB could overexpress adhesion and chimoattractant molecules, leading to the entry of CD8^+^ T cells into the CNS. *In situ*, CD8^+^ T cells could be reactivated by resident APC presenting target Ag(s), unknown for now. This could lead to the clonal expansion of CD8^+^ T cells along with the secretion of proinflammatory molecules. Finally, in this step, CD8^+^ T cells could be able to mediate damage to resident cells and axons potentially by the recognition of CNS derived peptides. Ag: antigen; GZM-B: granzyme-B; BBB: blood–brain barrier; CNS: central nervous system; MS: multiple sclerosis; EBV: Epstein–Barr virus; APC: antigen-presenting cell; TCR: T-cell receptor; MCAM: melanoma cell adhesion molecule; VCAM-1: vascular cell adhesion molecule 1; DC: dendritic cell; MHC-I: major histocompatibility complex I; CCR2: C–C chemokine receptor type 2; CCL2: C–C chemokine ligand 2; IFNγ: interferon γ; IL-17: interleukin-17.

To conclude, it appears clear to us that CD8^+^ T cells are involved in the pathophysiology of MS, in particular as potent effectors for CNS damage. However, other cell subsets, including CD4^+^ T cells, are likely to act in synergy to trigger the disease, probably by giving aid to pathogenic CD8^+^ T cells. More studies are needed to decipher the exact steps involving CD8^+^ T cells in the disease. Indeed, numerous questions remain unanswered: how and why CD8^+^ T cells get activated/reactivated in MS patients; how they cross the BBB; what the target antigen(s) is (are); how they mediate damage *in situ*. Focusing on these different questions and mechanisms is essential in order to develop effective therapeutic approaches (Figure 2).

## Conflict of Interest Statement

The authors declare that the research was conducted in the absence of any commercial or financial relationships that could be construed as a potential conflict of interest.

## Abbreviations

BBB, blood–brain barrier; CNS, central nervous system; CSF, cerebrospinal fluid; EAE, experimental autoimmune encephalomyelitis; EBV, Epstein–Barr virus; HLA, human leukocyte antigen; HV, healthy volunteers; MAIT, mucosal-associated invariant T; MBP, myelin basic protein; MHC, major histocompatibility complex; MRI, magnetic resonance imaging; MS, multiple sclerosis; NAWM, normal-appearing white matter; TCR, T-cell receptor.
